# Identification and immunoassay of biomarkers associated with T cell exhaustion in systemic lupus erythematosus

**DOI:** 10.3389/fimmu.2025.1476575

**Published:** 2025-03-26

**Authors:** Yiqing Zheng, Hejun Li, Yanqing Wang, Lixin Huang, Ling Chen, Shunping Lin, Shuhuan Lin

**Affiliations:** Department of Rheumatology, Fujian Medical University Union Hospital, Fuzhou, China

**Keywords:** systemic lupus erythematosus, T cell exhaustion, biomarkers, type I interferon, chemical compounds

## Abstract

**Background:**

Systemic lupus erythematosus (SLE) is an autoimmune disease with unclear etiology. T cell exhaustion (TEX) suppresses the immune response and can be a potential therapeutic strategy for autoimmune diseases. Therefore, this study primarily investigated the mechanism by which TEX influences SLE, offering a novel target for its treatment.

**Methods:**

GSE72326 and GSE81622 were utilized in this study. TEX related genes (TEX-RGs) were obtained from the published literature. Differentially expressed genes (DEGs) were obtained through differential expression analysis. Subsequently, candidate genes were selected by overlapping DEGs and TEX-RGs. These candidate genes underwent protein-protein interactions (PPIs) analysis for further screening. Machine learning was applied to identify candidate key genes from the PPI-identified genes. The candidate key genes exhibiting an area under the receiver operating characteristic (ROC) curve (AUC) greater than 0.7, along with consistent expression trends and significant differences in GSE72326 and GSE81622 were defined as biomarkers. Additionally, enrichment analysis, immune infiltration analysis, chemical compounds prediction and molecular docking were carried out. Importantly, the biomarkers were validated for expression by reverse transcription-quantitative polymerase chain reaction (RT-qPCR).

**Results:**

The biomarkers MX1, LY6E, IFI44 and OASL were screened by overlapping 327 DEGs and 1,408 TEX-RGs. Gene set enrichment analysis (GSEA) showed that there was a significant positive correlation between the expression of these biomarkers and immune-related pathways, such as the NOD-like receptor signaling pathway, Toll-like receptor signaling pathway and RIG-I-like receptor signaling pathway significant positive correlation. The immune infiltration of 8 types of immune cells differed significantly in SLE. Naive B cells, resting memory CD4 T cells and resting NK cells were significantly down-regulated in the SLE group. 4 biomarkers showed the highest correlation with resting memory CD4 T cells. Bisphenol A targeted OASL and LY6E, whereas acetaminophen targeted IFI44 and MX1.The binding activity between the biomarkers and the chemical compounds targeting them was very strong. Finally, RT-qPCR expression of MX1, LY6E, IFI44 and OASL was consistent with the results of the dataset.

**Conclusion:**

MX1, LY6E, IFI44 and OASL were identified as biomarkers related to TEX in SLE. These biomarkers could be detected in the blood for early diagnosis of the disease or to monitor the efficacy of the disease treatment, thus providing a new target for the management of SLE.

## 1 Introduction

Systemic lupus erythematosus (SLE) remains a poorly understood multifactorial autoimmune disease, with genetic, environmental, and hormonal factors believed to contribute to its etiology (1). The clinical manifestations of SLE are diverse. Due to the complexity of the clinical manifestations and the lack of specific diagnostic criteria, the diagnosis and treatment of SLE are extremely difficult (2). SLE patients often exhibit T lymphocytopenia, reduced inhibitory T cell function, and hyperplasia of B cells, resulting in the production of large numbers of autoantibodies against ds-DNA and other nuclear antigens, which subsequently trigger immune complex-induced inflammatory responses in various organs. In recent years, interference with B-cell activation targeting B-cell stimulators (BAFF/BLyS) or B-cell monoclonal antibodies targeting CD20 has been used to treat SLE, and CAR-T (Chimeric Antigen Receptor T) cell immunotherapy has also enabled SLE patients to achieve rapid remission with high safety (3). B cell depletion has emerged as an effective treatment strategy for SLE. However, clinical observations reveal that a subset of patients does not respond favorably to B-cell depletion therapy, with 52-week response rates for belimumab and rituximab reported as 53.8% and 29.6% respectively (4, 5), and for telitacicept the 52-week response rate was 82.6% (6). Although cellular therapy has had some success in patients with SLE, the presence of extremely complex immune pathways in patients with SLE has necessitated the search for new ways to treat SLE.

T cell exhaustion (TEX), commonly referred to as CD8+T cell exhaustion, represents a state of diminished functionality within T lymphocytes (7). T lymphocytes have a variety of biological functions, ranging from the direct elimination of target cells, assisting or inhibiting B cells to produce antibodies, responding to specific antigens and mitogen, such as producing cytokines, etc. Central to the body’s defense against disease infections and tumor formation, T cells orchestrate cellular immunity, which operates through two primary mechanisms: specific binding and destruction of target cell membranes leading to direct cell killing, and the release of lymphocytokines that amplify and potentiate the immune response. TEX arises from prolonged antigenic stimulation, often triggered by the persistent presence of invasive pathogens in chronic infection states or the exhaustion of T cells resulting from chronic antigenic exposure in tumorous environments (8). Blanco et al. (9) demonstrated that CD8+T cells exhibited a more prominent cytotoxic phenotype and functions during SLE onset than during remission. Furthermore, the frequency of such cells was correlated with SLE Disease Activity Index (SLEDAI) score. In the study conducted by Winchester et al. (10). tubulointerstitial nephritis associated with CD8+T cell infiltration was identified as a risk factor for the progression of lupus nephritis (LN). This is consistent with the previous research results of Couzi et al. (11)., which indicated that CD8+T cell can also generate CD4-CD8-double negative (DN) T cells in an inflammatory environment, subsequently secrete IL-17, infiltrate the kidney and induce tissue damage (12). Collectively, these studies suggest a pivotal role for CD8+T cells in the pathogenesis of SLE. However, despite studies indicating a relationship between TEX and systemic autoimmune diseases, the exact therapeutic mechanism of TEX in SLE remains relatively scarce.

To screen biomarkers for SLE, we performed differential expression profiling, machine learning techniques, enrichment analysis, immune infiltration assessment, regulatory network construction, chemical compounds prediction, molecular docking simulation, and expression validation on datasets and genes from the public database. These findings offer promising potential targets for clinical diagnosis of SLE and prognostic predictions, while also providing a theoretical foundation for further elucidating the intricate mechanisms underlying SLE.

## 2 Materials and methods

### 2.1 Data extraction

GSE72326 (platform: GPL10558) and GSE81622 (platform: GPL10558) were acquired from the public database-Gene Expression Omnibus (GEO) database (http://www.ncbi.nlm.nih.gov/geo/) containing a rich set of genes associated with expression in patients with SLE. The GSE72326 dataset, comprising 238 samples, was employed as the training set. This study encompassed a total of 177 blood samples (SLE: Control = 157: 20). The validation set GSE81622 consisted of 30 SLE blood samples and 25 control blood samples. Additionally, 1408 TEX related genes (TEX-RGs) were obtained from the published literature (13).

### 2.2 Differential expression analysis

Differentially expressed genes (DEGs) were obtained through differential expression analysis (SLE vs Control) in training set using limma package (version 3.56.2) (14), with screening criteria of |log_2_fold change (FC)| > 0.5 and p.adjust < 0.05. Volcano plots and heatmaps were drawn to show the expression of DEGs using ggplot2 package (version 3.4.2) (15) and circlize package (version 0.4.15) (16), respectively. Among them, the heat maps exclusively depicted the top 10 DEGs with the most significant up- and down-regulation.

### 2.3 Functional and protein-protein interaction analysis

Candidate genes were obtained by overlapping DEGs and TEX-RGs. The clusterProfiler package (version 4.8.2) (17) was employed to perform enrichment analysis of candidate genes containing Gene Ontology (GO) and Kyoto Encyclopedia of Genes and Genomes (KEGG), aiming to investigate the shared functions and signaling pathways among these candidate genes (p < 0.05). GO analysis included cellular component (CC), molecular function (MF) and biological process (BP). The GOplot package (version 1.0.2) (18) was used to draw a chord plot to show the top 8 significantly enriched pathways. In order to explore whether there was an interaction between candidate genes, a PPI network was constructed through STRING database (https://cn.string-db.org/) (confidence > 0.4). Subsequently, MCODE plug-in in Cytoscape software (version 3.9.1) (19) was used to identify important candidate genes in PPI network for subsequent analysis.

### 2.4 Identification of biomarkers

Least absolute shrinkage and selection operator (LASSO) regression was a regularization method for linear regression. It added an L1-paradigm penalty term to the objective function on the basis of ordinary least squares regression, which served to shrink the regression coefficients, making the coefficients of some unimportant independent variables converge to 0, so as to achieve the purpose of variable selection (20). Support vector machine-recursive feature elimination (SVM-RFE) was a powerful classification algorithm whose goal was to find a hyperplane such that data points of different categories were maximally spaced on either side of this hyperplane (21). It measured the importance of features by calculating the squared paradigm of the weight vector corresponding to each feature; the smaller the paradigm, the less important the feature. The key candidate genes were further screened by LASSO and SVM-RFE methods using glmnet package (version 4.1-7) (22) and e1071 package (version 1.7-13), respectively. Subsequently, these two parts of obtained genes were intersected to gain candidate key genes. Furthermore, the receiver operating characteristic (ROC) curve analysis and expression verification of candidate key genes were performed in GSE72326 and GSE81622. The ROC curve (Receiver Operating Characteristic curve) was a tool for evaluating the performance of a classification model by plotting the True Positive Rate (TPR) and False Positive Rate (FPR) at different thresholds. The closer the AUC value was to 1, the better the model performance (23). The candidate key genes exhibiting an area under the ROC curve (AUC) greater than 0.7, along with consistent expression trends and significant differences in GSE72326 and GSE81622 were defined as biomarkers. Additionally, Spearman analysis of biomarkers was conducted using psych package (version 2.3.6) (24). Spearman correlation analysis was a non-parametric method of correlation analysis which was used to measure the strength and direction of a monotonic relationship between biomarkers.

### 2.5 Gene-gene interaction and gene set enrichment analysis

A GGI network was constructed by GeneMANIA database (https://genemania.org/) to investigate the genes and functions associated with biomarkers. The TOP 20 genes most relevant to biomarkers, as well as the top 7 pathways involved in biomarkers were selected for presentation. Additionally, GSEA was used to assess whether a pre-defined set of genes was significantly differentially enriched between two sets of samples to further explore the functional implications of the biomarker (25). Firstly, Spearman correlation analysis was performed between each biomarker and all genes in GSE72326 using psych package (version 2.3.6). Secondly, the results were sorted based on the correlation coefficient. Subsequently, GSEA enrichment analysis was carried out using clusterProfiler package (version 4.8.2), utilizing the KEGG background gene set from Molecular Signatures Database (MSigDB) (https://www.gsea-msigdb.org). The screening criteria were |NES| > 1 and p.adjust < 0.05.

### 2.6 Immune infiltration analysis

Immune infiltration refers to the process by which immune cells infiltrate into specific tissues or organs. This process exemplifies the response and regulation of the body’s immune system towards the local microenvironment. Therefore, we further investigate the infiltration of immune cells in the blood tissues of SLE patients and a control group, aiming to uncover the enrichment patterns of immune cells during the progression of SLE. The proportion of 22 immune cells in GSE72326 was calculated using CIBERSORT (version 1.03) (26) algorithm based on the LM22 gene set. Immune cells with immune infiltration of 0 were removed, resulting in 19 types for subsequent analysis. A histogram was generated using ggplot2 package (version 3.4.2) to visualize immune cell infiltration. The infiltration differences of 19 immune cells between SLE and control groups were compared through Wilcoxon rank sum test. Finally, Spearman correlation analysis was performed on biomarkers and differential immune cells followed by visualization using a lollipop map created with ggplot2 package (version 3.4.2).

### 2.7 Construction of regulatory networks

The miRNAs targeting biomarkers were predicted using starBase database (http://starbase.sysu.edu.cn/ and microRNA target prediction database (miRDB) (http://www.mirdb.org/), respectively. Subsequently, these two sets of miRNAs were overlapped to obtain key miRNAs. In addition, lncRNAs targeting key miRNAs were predicted through miRNet database (https://www.mirnet.ca/) and starBase database (http://starbase.sysu.edu.cn/). The lncRNAs obtained from these two databases were intersected to obtain key lncRNAs. Based on the identified key miRNAs, key lncRNAs and biomarkers, a competitive endogenous RNA (ceRNA) network was constructed. To further explore the upstream regulatory mechanism of biomarkers in diseases, transcription factors (TFs) that can regulate biomarkers were predicted based on JASPAR database (http://jaspar.genereg.net/) from NetworkAnalyst platform (https://www.networkanalyst.ca/). Furthermore, a mRNA-TFs network were conducted.

### 2.8 Chemical compounds prediction and molecular docking analysis

The Comparative Toxicogenomics Database (CTD) (http://ctdbase.org/) was utilized for the prediction of chemical compounds targeting biomarkers. Compounds with a reference count greater than 2 were selected. Furthermore, Cytoscape software (version 3.9.1) was employed to construct a network illustrating the relationship between chemical compounds and biomarkers. To further validate the role of biomarkers in the treatment process, we conducted molecular docking analysis between biomarkers and predicted chemical compounds. The protein structure corresponding to the biomarkers was retrieved from Protein Data Bank (PDB) database (https://www.rcsb.org/), while the AlphaFold prediction structure was obtained from AlphaFold (https://alphafold.ebi.ac.uk/). The molecular structure of ligand (chemical compounds obtained through prediction) was acquired from PubChem database (https://pubchem.ncbi.nlm.nih.gov), followed by molecular docking of the biomarker’s protein structure with its respective ligand using Autodock software. It was widely accepted that a more stable conformation of ligand-receptor binding corresponds to lower required binding free energy. A molecular binding free energy ≤ -5.0 kcal/mol indicated superior binding activity (27).

### 2.9 Expression assessment of biomarkers

The blood samples from the 6 SLE patients and 6 healthy individuals were gained in Fujian Medical University Union Hospital in China. All of the patients with SLE have positive ANA and fulfilled the Systemic Lupus International Collaborating Clinics (SLICC) 2012 SLE classification criteria (28). In addition, all included patients (SLE and Control) did not receive any immunomodulators or hormonal therapy and immunosuppressive drugs. Patients with acute and chronic infections, pregnancy and malignancies were excluded. We also collected the patient’s (SLE and Control) age, sex, SLEDAI-2000 score (29), and clinical laboratory test results such as Antiphospholipid antibody, complement, anti-dsDNA, and anti-Smith antibody (**Table 1**). These blood samples were utilized for reverse transcription quantitative polymerase chain reaction (RT-qPCR) analysis. This study was approved by the ethics board of Fujian Medical University Union Hospital (Ethical number: 2023KY234). All patients (SLE and Control) had signed an informed consent form. Following the manufacturer’s instructions, TRIzol (Ambion, Austin, USA) was utilized to extract the total RNA of 12 samples. Following the manufacturer’s instructions, the SureScript-First-strand-cDNA-synthesis-kit (Servicebio, Wuhan, China) was employed to reverse transcribe whole RNA to cDNA. The 2xUniversal Blue SYBR Green qPCR Master Mix (Servicebio, Wuhan, China) was utilized to conduct the qRT-PCR. **Supplementary Table 1** displayed the PCR primer sequences. As an internal reference gene, GAPDH was used. Candidate biomarkers’ expression was calculated using the 2^−ΔΔCt^ technique (30).

**Table 1:** Clinical and laboratory characteristics of patients with SLE and control.

Characteristics	SLE (n=6)	Control (n=6)
Females	6	6
Age, years (median, IQR)	28.0 (20.8-38.0)	20.0 (20.0-38.8)
Acute/Subacute cutaneous lupus	2	
Non-scarring alopecia	1	
Arthritis	2	
Pleural or pericardial effusion	4	
Renal involvement	2	
Hematologic involvement	5	
SLEDAI-2000 score (mean ± SD)	13.50 ± 4.85	
C3, mg/dL (mean ± SD)	0.34 ± 0.18	
C4, mg/dL (median, IQR)	0.03 (0.02-0.08)	
Anti-dsDNA antibody (%)	6 (100.0)	
Anti-Sm antibody (%)	3 (50.0)	
Antiphospholipid antibody (%)	1 (16.7)	

### 2.10 Statistical analysis

R software (version 4.2.1) was used to process and analyze the data. The Wilcoxon rank sum test was employed to assess the differences between different groups. Also correction was performed using the Benjamini-Hochberg (BH). The p value less than 0.05 was considered statistically significant.

## 3 Results

### 3.1 A total of 13 important candidate genes were identified in SLE

**Figure 1:**
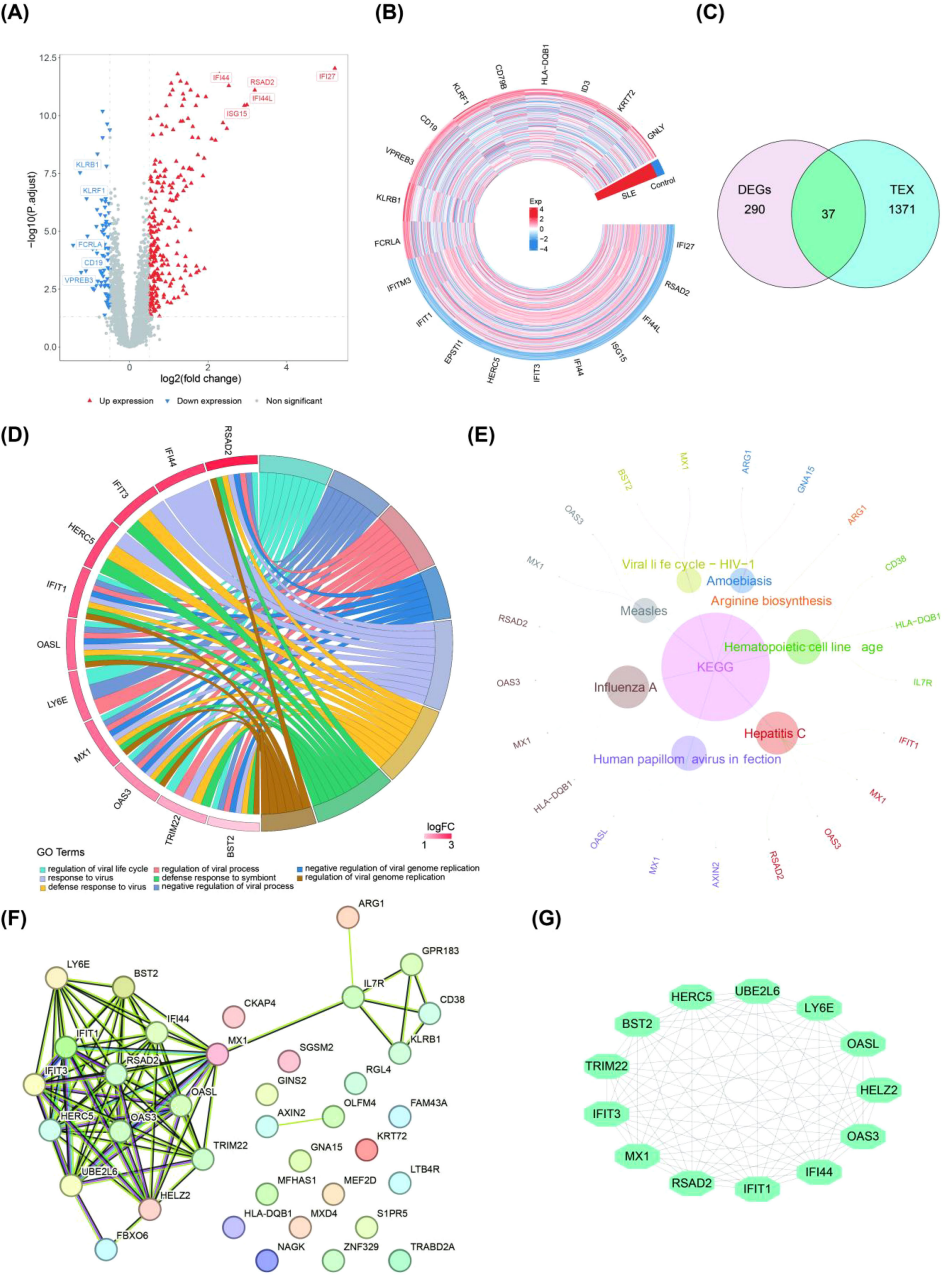
Differential expression analysis of important candidate genes (SLE patients vs. healthy controls). **(A, B)** Volcano and heat maps of DEGs obtained by differential analysis based on the public database dataset GSE72326 (The top 10 genes showing the most significant up- and down-regulation). **(C)** Venn diagram of the overlapped candidate genes. **(D, E)** GO analysis and KEGG analysis of candidate genes. **(F, G)** The PPI network in candidate genes and the most significant module (GO, gene ontology; KEGG, Kyoto Encyclope dia of Genes and Genomes; PPI, protein–protein interaction).

The SLE group and the control group in the dataset GSE72326 were analyzed by differential expression analysis to obtain 327 DEGs of which 243 genes were expressed significantly high expression and 84 genes were expressed significantly lower expression (**Figures 1A, B**). Then, 37 candidate genes were obtained by taking the intersection of 327 DEGs and 1,408 TEX-RGs in the literature (**Figure 1C**). Enrichment analysis demonstrated that these candidate genes were enriched to 292 GO terms (243 BP, 23 CC, and 23 MF) and 8 KEGG pathways (**Figures 1D, E**). These enriched pathways included negative regulation of viral genome replication, defense response to symbiont, hematopoietic cell lineage, viral life cycle-HIV-1 and so on. Through PPI analysis of 37 candidate genes, we found that 13 important candidate genes had strong interaction with each other. Among them, LY6E interacted with BST2, IFIT3, MX1, RSAD2, IFIT1, IFI44, OAS3, and OASL (**Figures 1F, G**).

### 3.2 MX1, LY6E, IFI44 and OASL were identified as biomarkers related to TEX on SLE

**Figure 2:**
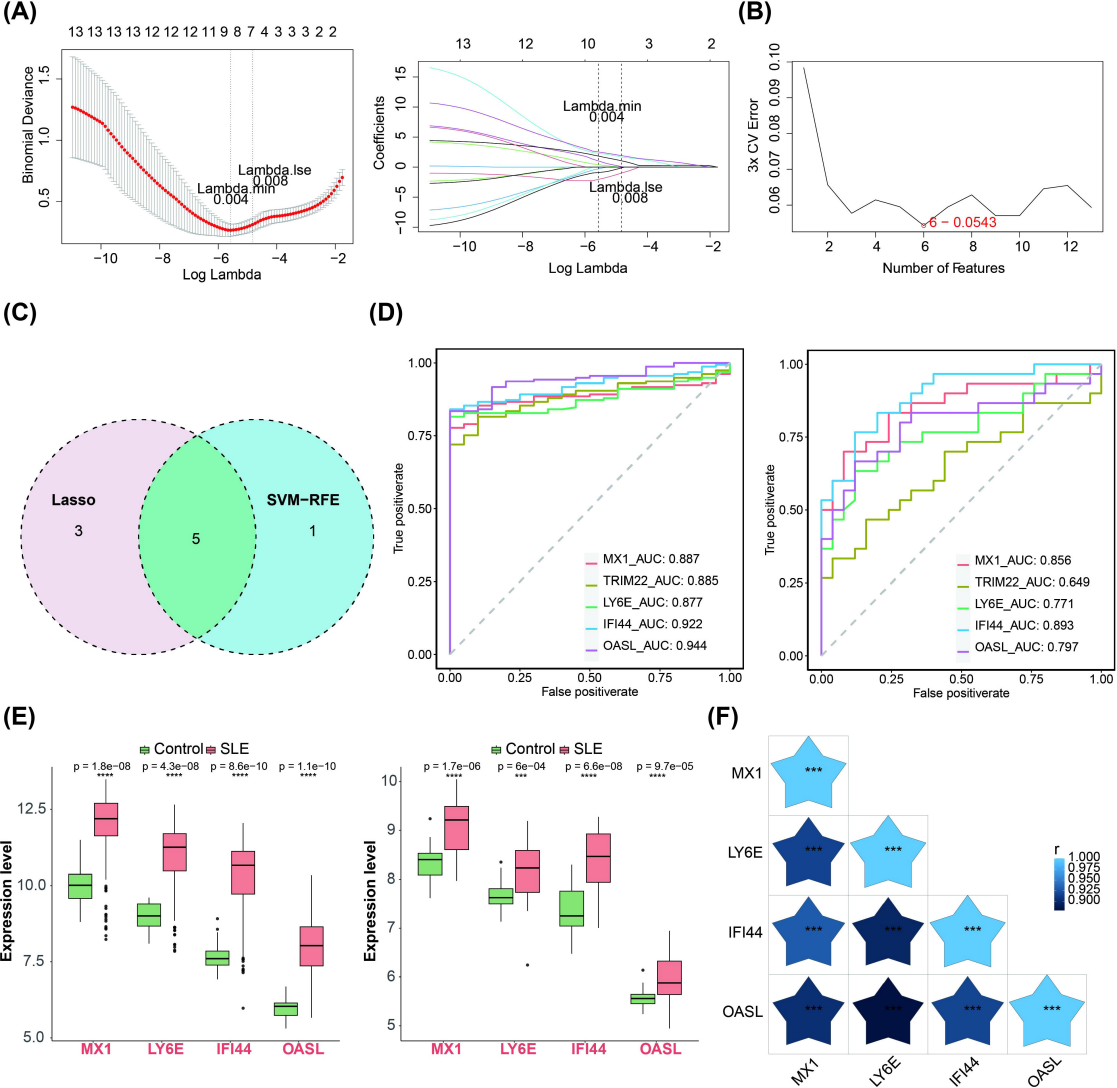
Machine learning screening for biomarkers. **(A–C)** LASSO regression and SVM-RFE algorithms for screening key candidate genes. The horizontal coordinate in the left panel was the log(lambda) value and the vertical coordinate is the degree of freedom, which represents the cross-validation error. In the right graph the horizontal coordinates were log(lambda) and the vertical coordinates are the coefficients of the genes. The dashed position was the position with the smallest cross-validation error, and the corresponding horizontal coordinate log (Lambda) was determined based on this position (Lambda.min), and the upper horizontal coordinate shows the number of characterized genes to find the optimal log (Lambda) value. **(D)** Diagnostic performance of 4 biomarkers in SLE diagnosis (AUC > 0.7, training set GSE72326 and validation set GSE81622). **(E)** Expression validation of 4 biomarkers. The left figure showed the training set GSE72326 and the right figure showed the validation set GSE81622 (Significance: ns, P > 0.05, *P < 0.05, **P < 0.01, ***P < 0.001, ****P < 0.0001). **(F)** Heatmap of 4 biomarkers correlations (The shade of the blue pentagram in the graph represents the degree of relevance. The lighter the colour the higher the correlation. Significance: ns, p > 0.05, *P < 0.05, **P < 0.01, ***P < 0.001, ****P < 0.0001). The figure was obtained by analysing the dataset based on the public database.

We subjected the 13 important candidate genes obtained to LASSO and SVM-RFE analyses. The candidate key genes, namely MX1, TRIM22, LY6E, IFI44 and OASL, were identified through the integration of 8 genes from LASSO analysis and 6 genes from SVM-RFE analysis (**Figures 2A–C**). The ROC curve analysis revealed that the AUC values of MX1, LY6E, IFI44, and OASL in both GSE72326 and GSE81622 exceeded 0.7, indicating their strong discriminatory power on SLE (**Figure 2D**). Moreover, these candidate key genes exhibited consistent expression patterns across both GSE72326 and GSE81622 and were significantly up-regulated in SLE group (**Figure 2E**). Consequently, MX1, LY6E, IFI44, and OASL were identified as biomarkers associated with TEX on SLE. Additionally, there was a significant positive correlation among these four biomarkers (**Figure 2F**).

### 3.3 The expression of biomarkers was significantly positively correlated with immune related pathways

**Figure 3:**
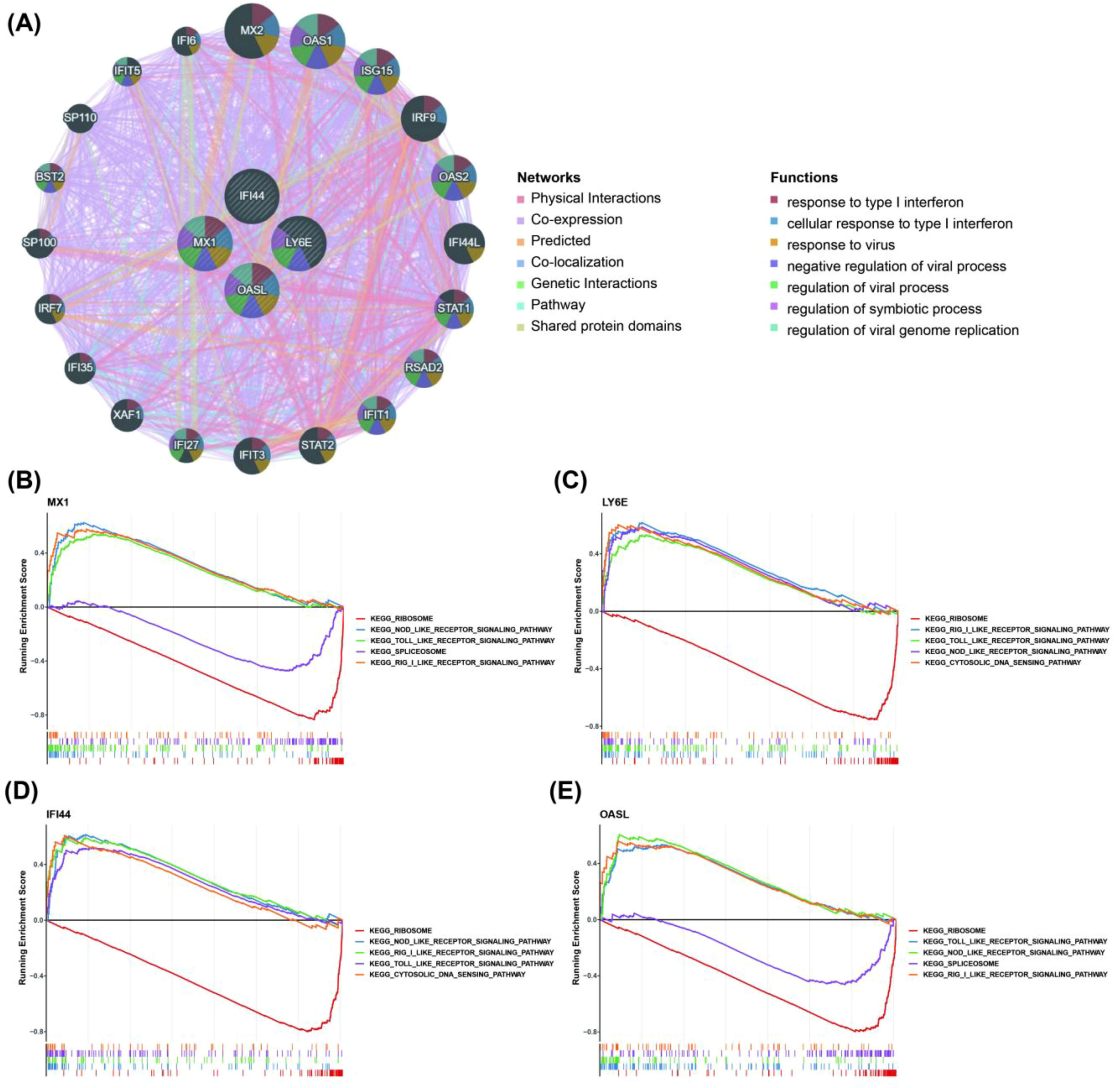
Relationship between biomarkers and immune related pathways. **(A)** The GGI network for biomarkers (Demonstrate the top 20 genes in terms of correlation with 4 biomarkers and the top 7 pathways in terms of significance. GGI, gene–gene interaction). **(B–E)** GSEA enrichment analysis of biomarkers in the training set GSE72326 (Display the entries with the top 5 P-adjust values). The horizontal axis was the sorted genes and the vertical axis was the corresponding Running ES. The peak in the line graph was the enrichment score of the gene set of this pathway; the lower part of the line marks the genes that were located under this gene set.

We observed that these biomarkers were associated with OAS1, ISG15, IRF9, RSAD2, IFIT1 and other genes through constructing a GGI network. The pathways in which they were implicated encompassed response to type I interferon (IFN-I), cellular response to IFN-I, regulation of symbiotic processes and more (**Figure 3A**). Furthermore, GSEA analysis revealed significant enrichment of MX1, LY6E, IFI44, and OASL in 17, 11, 10, and 19 pathways respectively. The enrichment results of ranking top 5 from small to large according to p-adjust value were selected for display. Additionally, MX1, LY6E, IFI44 and OASL were significantly co-enriched in NOD-like receptor (NLR) signaling pathway, Toll- like receptor (TLR) signaling pathway and RIG-I-like receptor (RLR) signaling pathway (**Figures 3B–E**).

### 3.4 There was the most significant correlation between biomarkers and resting memory CD4 T cells

**Figure 4:**
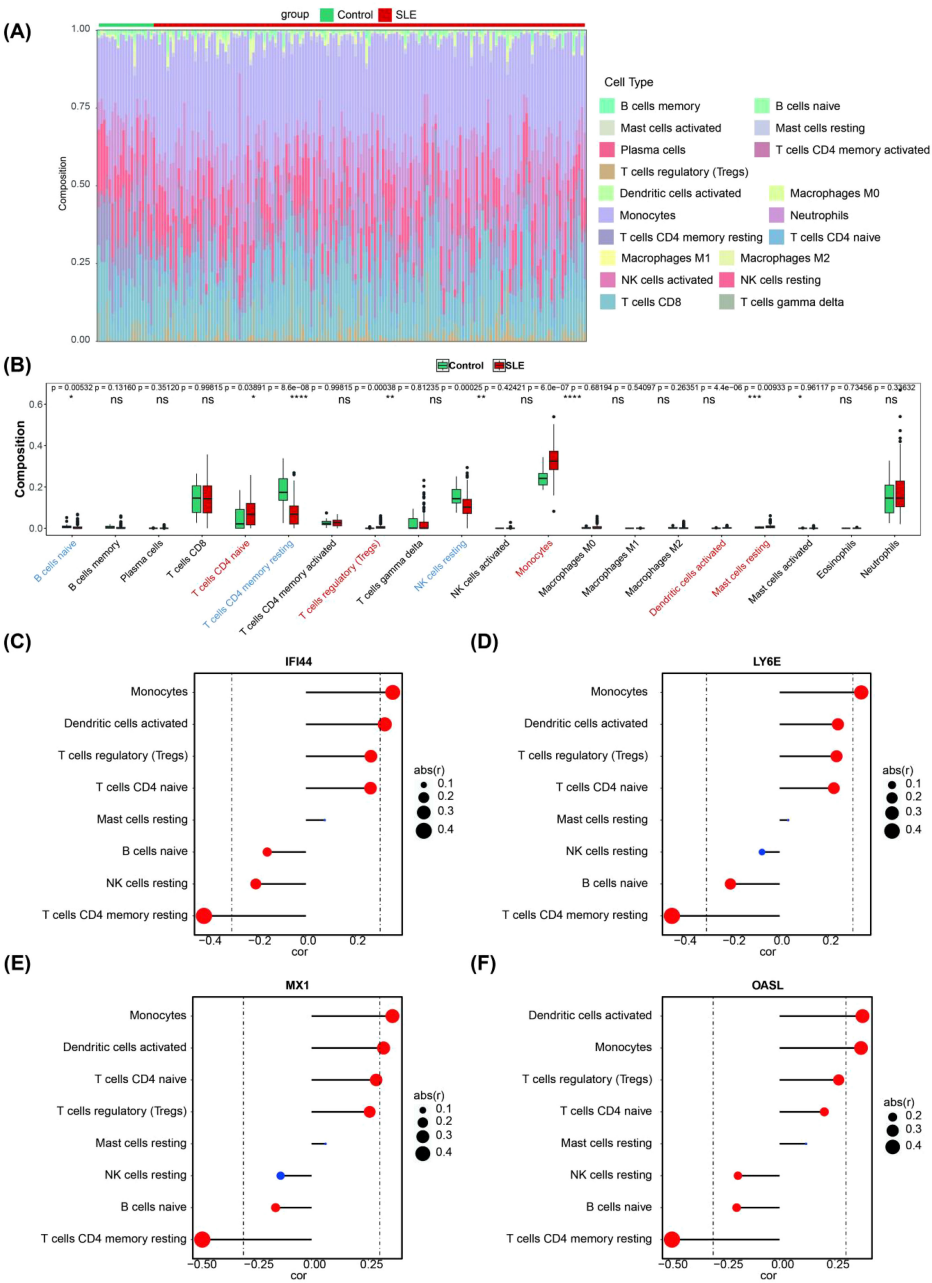
Immune infiltration analysis. **(A)** The landscape of 19 types of immune cell infiltration in the GSE72326 dataset. **(B)** Boxplot of the proportion of 19 types of immune cells in the GSE72326 dataset (Significance: ns, P > 0.05, *P < 0.05, **P < 0.01, ***P < 0.001, ****P < 0.0001). **(C–F)** Correlation between the expression levels of biomarkers and the immune infiltration level of differential immune cell (The size of the circle indicates correlation, the larger the correlation the better; the colour indicates significance, red means significant and blue means not significant).

The infiltration abundance of 19 immune cell types in the blood tissues of both the control group and the SLE group is shown in **Figure 4A**, where infiltration abundance refers to the relative number or proportion of immune cells within the blood tissue. These differential immune cells included naive B cells, naive CD4 T cells, resting memory CD4 T cells, regulatory T (Tregs) cells, resting NK cells, monocytes, activated dendritic cells and resting mast cells. Among these differential immune cells, naive B cells, resting memory CD4 T cells and resting NK cells exhibited significant down-regulation in SLE compared to other differential immune cells (**Figure 4B**). Importantly, 4 biomarkers showed the most significant correlation with resting memory CD4 T cells (**Figures 4C–F**). Memory CD4 cells are a special class of T lymphocytes that are activated and differentiated from the primary immune response of CD4 T cells. They play a crucial role in maintaining immune memory and preventing reinfection by pathogens (31).

### 3.5 Construction of ceRNA and mRNA-TFs regulatory networks for biomarkers

**Figure 5:**
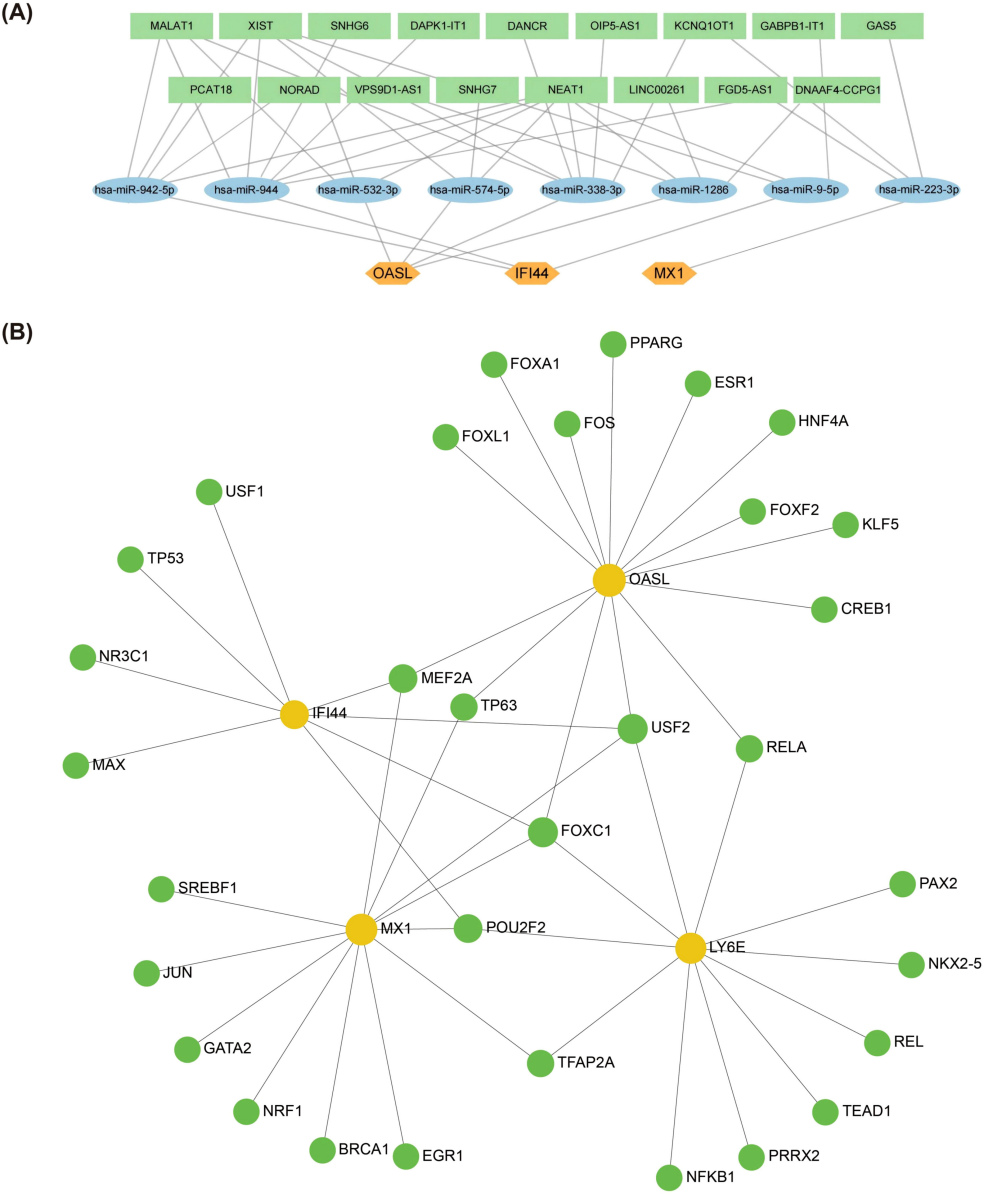
Biomarkers regulatory network analysis. **(A)** Potential impact of ceRNA on biomarkers expression through interaction with miRNAs. This figure predicted the corresponding intersecting miRNAs for the key genes in the starBase database (http://starbase.sysu.edu.cn/) and in the miRDB database (http://www.mirdb.org/) **(B)** Regulatory networks of TF-mRNAs and biomarkers. The figure was in the NetworkAnalyst platform (https://www.networkanalyst.ca/) based on the JASPAR database predicting TFs that can regulate key genes (Orange circles are mRNA and green circles are TF. TFs, Transcription Factors).

Firstly, a total of 8 key miRNAs (targeting 3 biomarkers) were identified by integrating 150 miRNAs from starBase database and 114 miRNAs from miRDB database. Subsequently, based on these 8 key miRNAs, we further identified 17 key lncRNAs by intersecting 267 lncRNAs from miRNet database and 48 lncRNAs from starBase database. Consequently, utilizing these 3 biomarkers along with the aforementioned 8 key miRNAs and 17 key lncRNAs, we constructed a ceRNA network. The regulation of OASL by NEAT1 in this network involved hsa-miR-532-3p, hsa-miR-574-5p, hsa-miR-338-3p and hsa-miR-1286. IFI44 was regulated by NEAT1 through hsa-miR-942-5p, hsa-miR-944, and hsa-miR9-5p, while MX1 was regulated by GAS5 via hsa-miR-223-3p (**Figure 5A**). Additionally, a total of 32 TFs regulating biomarkers were obtained. In the constructed TFs-mRNA regulatory network, IFI44, MX1, LY6E and OASL were simultaneously regulated by FOXC1 and USF2 (**Figure 5B**).

### 3.6 To predict the binding activity between biomarkers and chemical compounds that target them

**Figure 6:**
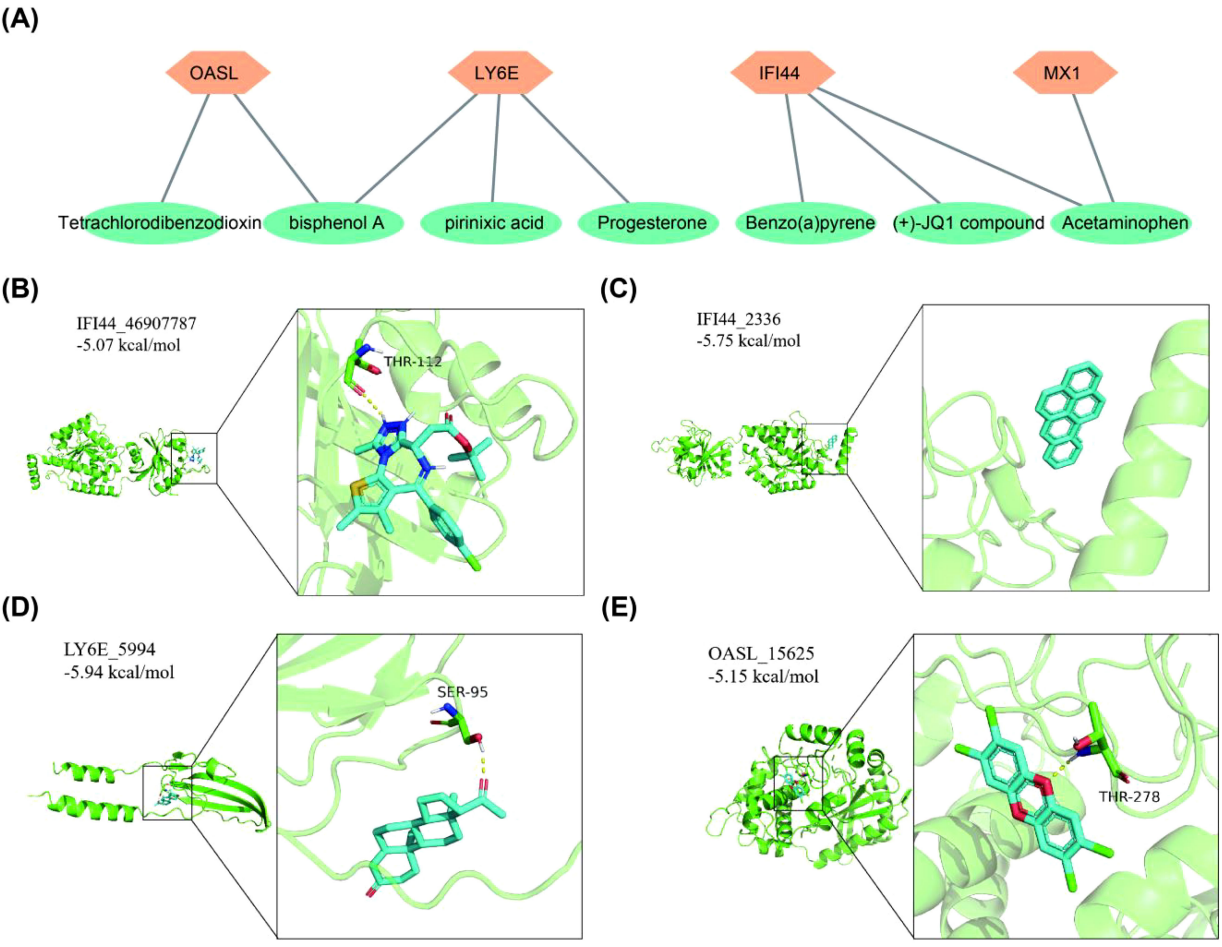
Chemical compounds prediction and molecular docking. **(A)** Biomarkers corresponds to target chemical compounds network diagram. **(B)** IFI44-(+)-JQ1 compound. **(C)** IFI44-Benzo(a)pyrene. **(D)** LY6E-Progesterone. **(E)** OASL-Tetrachlorodibenzodioxin.

It was predicted that there were 1, 3, 3, 2 kinds of chemical compounds targeting MX1, LY6E, IFI44 and OASL, respectively. Among these chemical compounds, bisphenol A (BPA) was found to target both OASL and LY6E, while acetylaminophen targeted both IFI44 and MX1 (**Figure 6A**). Furthermore, the molecular docking results demonstrated that a hydrogen bond (-5.07 kcal/mol) formed between the amino acid residue THR-112 of (+)-JQ1 compound and IFI44. There may be a non-hydrogen bond interaction (-5.75 kcal/mol) with benzo(a)pyrene (BaP). Additionally, a hydrogen bond interaction (-5.94 kcal/mol) was observed between the amino acid residue SER-95 of progesterone and LY6E. Another hydrogen bond interaction was identified between the amino acid residue THR-278 of tetrachlorodibenzodioxin and OASL (-5.18 kcal/mol) (**Table 2**, **Figures 6B–E**).

**Table 2:** Docking results of potential targets with chemical compounds.

Target	Chemical compounds	Compound CID	Binding energy (kcal/mol)
IFI44	(+)-JQ1 compound	46907787	-5.07
	Acetaminophen	1983	-4.38
	Benzo(a)pyrene	2336	-5.75
LY6E	bisphenol A	6623	-4.48
	pirinixic acid	5694	-4.82
	Progesterone	5994	-5.94
MX1	Acetaminophen	1983	-4.08
OASL	bisphenol A	6623	-4.63
	Tetrachlorodibenzodioxin	15625	-5.18

### 3.7 The RT-qPCR results were consistent with those of the dataset

**Figure 7:**
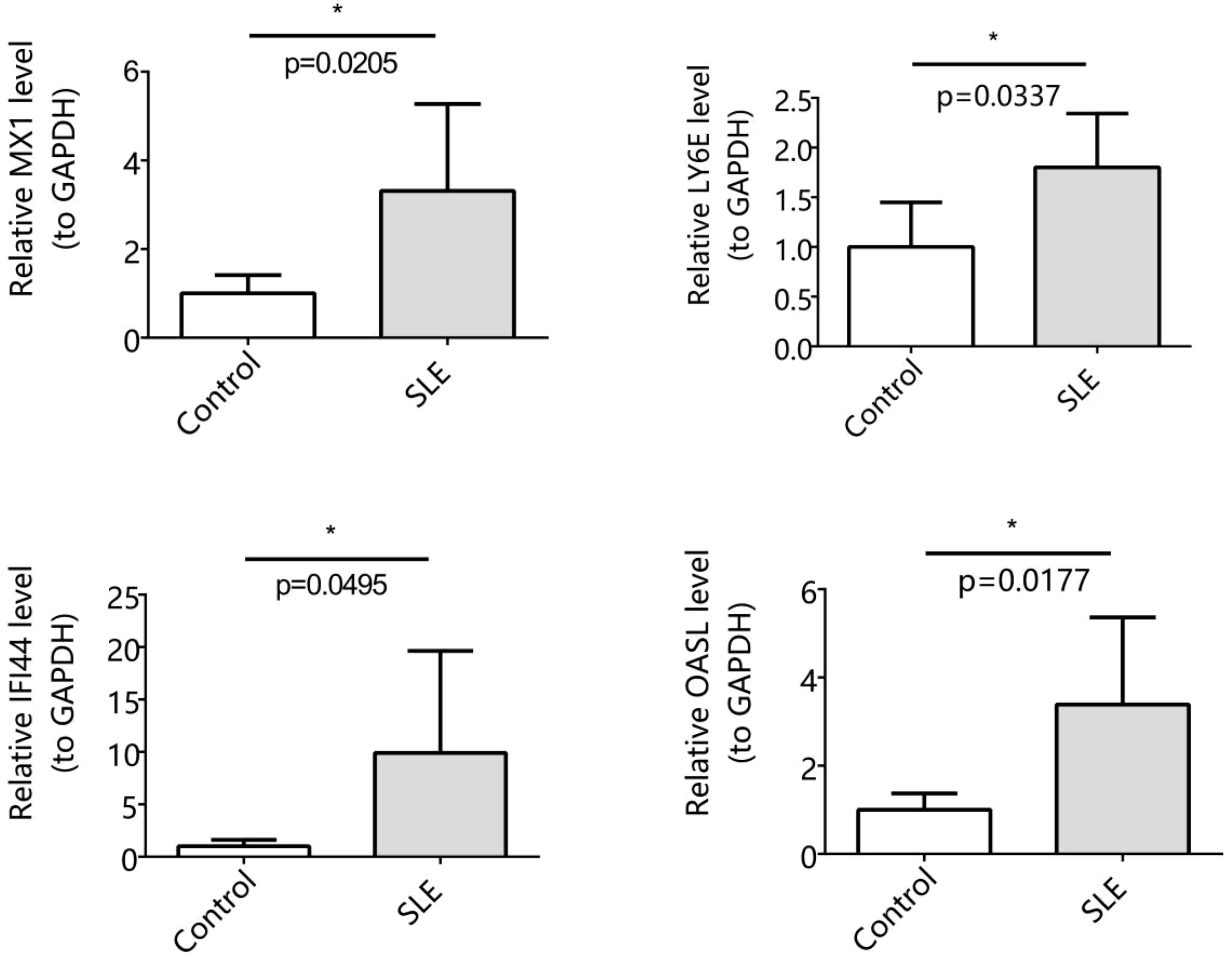
Expression levels of 4 biomarkers in SLE and control (Significance: *P < 0.05).

The expression of MX1, LY6E, IFI44, and OASL was assessed using RT-qPCR analysis. Our findings revealed a significant up-regulation of MX1, LY6E, IFI44, and OASL in the SLE group compared to the control group (p < 0.05). Importantly, these results were consistent with those obtained from the dataset (**Figure 7**).

## 4 Discussion

TEX diminishes the immune response mediated by T cells, leading to decreased secretion of effector cytokines, poorer proliferative capacity, reduced persistence, and enhanced expression of cell surface receptors (e.g., PD-1 and CTLA-4 on CD 8+T cell surface) (32). And in autoimmune diseases, patients with TEX show a better prognosis (33, 34). Therefore, there is an urgent need to better understand the detailed mechanisms of TEX to provide new strategies for the treatment of SLE.

Previous studies have shown that MX1 is highly expressed in the glomeruli and that this gene is associated with renal fibrosis (35). Peripheral blood MX1 gene expression has been used as a sensitive biomarker for LN therapy (36). The immune systems of mice and humans share some similarities, yet they also exhibit numerous differences in physiological structure and function. Despite these variations, observing the production of specific antibodies and local inflammatory responses in the kidney of mice may offer insights that are more readily discernible (37). In experiments with lupus mice, the expression level of Ly-6A/E on splenic lymphocytes was positively correlated with disease severity (38). The expression of Ly6E in proximal convoluted renal tubules is elevated due to proteinuria (39), suggesting a potential role for LY6E in nephropathophysiology. OASL expression in PBMCs and CD19+ B cells is upregulated in patients with active SLE with renal disease compared with patients without clinical manifestations (40). IFI44, as a LN specific biomarker, can distinguish between patients with active and inactive LN (41). In our study, four biomarkers MX1, LY6E, OASL and IFI44 were screened, and they are ISGs (42). These biomarkers, which are all upregulated in SLE patients, are closely linked to the activity of SLE disease. This is consistent with previous studies (43–45). A previous study comparing lymphocyte subtypes in healthy and SLE patients by two-color immunofluorescence flow cytometry analysis showed a reduction in circulating CD8+ T cells in SLE patients (491(146-1136) VS 331 (92-1401), p < 0.01) (46), but there are no relevant studies at the genetic level. This is also the innovation of this study.

Interferon-stimulated genes (ISGs) are produced by induction of interferon (IFN), and the activities of IFN-Is are potentiated by hundreds of ISGs. ISGs include antimicrobial proteins, chemokines, cytokines and other immune mediators that induce recruitment of immune cells and inflammation. ISGs play diverse roles in many cellular processes such as migration, antigen processing and presentation, cellular activation, differentiation, mitosis and apoptosis (47). Previous *in vitro* experiments have shown that anti-LY6E antibody treatment leads to CD3ζ chain tyrosine phosphorylation and blocks TCR-mediated T cell apoptosis (48). It indirectly reflects the role of LY6E in T cell apoptosis. In a cytological study by Perez et al., it was found that SLE patients experienced a decrease in Naive CD4+ T cells and an expansion of cytotoxic GZMH+ CD8+ T cells, as well as increased expression of ISG signals. It is speculated that IFN-1 causes monocytes to explicitly express ISGs and inhibits the basis of CD4+ T cell export to lymphoid tissue, leading to the observed decrease in circulating naive CD4+ T cells (49). This is consistent with the clinically observed lymphocytopenia in SLE patients and our findings. In studies of COVID-19, strong infiltration of CD8 T cells into lung tissue and manifestations of TEX have been shown in patients with low local expression of ISG (50). Previous scholarly endeavors have established that a substantial quantity of endogenous DNA, present in the cytoplasm beyond the confines of the nucleus and mitochondria, possesses the capacity to activate the cGAS-STING signaling pathway (51). Upon activation, STING meticulously orchestrates the recruitment of TANK-binding kinase 1 (TBK1), which, in turn, activates and phosphorylates interferon regulatory factor 3 (IRF-3), thereby stimulating the production of IFN-I. These IFN-1s subsequently engage with the IFN-I receptor, igniting the JAK/STAT signaling cascade and prompting the transcription of ISGs (52). Notably, the STING gene itself is classified as an ISG, paving the way for a potential self-sustaining positive feedback loop (53). Recent research has further demonstrated that elevated STING expression in T cells triggers an enhanced response associated with the induction of IRF-3-dependent and p53-dependent pro-apoptotic genes, ultimately culminating in the programmed cell death or apoptosis of T cells (54). During chronic infections and perhaps some cancers, IFN-α/β and IFN-γ can remain elevated and induce the expression of IL-10, IDO, PD-L1, TIM-3, LAG-3 and other negative regulators of T cell responses (32, 55, 56). CXCL9, CXCL10 are primarily secreted by monocytes, endothelial cells, fibroblasts, and cancer cells in response to IFN-γ, CXCL10 can also be strongly induced by IFN-α/β (57), and may also play a role in TEX (32). IFN-α/β can also foster attrition of activated T cells via Fas/FasL-mediated T cell death and perhaps other mechanisms (58, 59). There is also evidence that high IFN-α/β signaling can promote terminal exhaustion by antagonizing the TEX progenitor pool through effects on the transcription factor T cell factor-1 (Tcf-1) (60). Chen et al. showed that chronic IFN-I stimulation disrupted lipid metabolism and REDOX balance in TEX cells, resulting in abnormal lipid accumulation and elevated oxidative stress, and these defects promoted lipid peroxidation, thereby enhancing TEX cell metabolism and directly contributing to the terminal exhaustion of CD8+ T cells. At the same time, *in vitro* studies showed that IFN-I treated TEX cells exhibit greater gene enrichment associated with TEX, including MX1, OSAL (61). Studies have also found that long-term exposure to IFN-I increases NAD+ consumption of CD8+ T cells, triggering mitochondrial changes in CD8+T cells, resulting in impaired mitochondrial respiration and reduced cell viability, and promoting CD8+T cell death after TCR reactivation (62). At the same time, IFN can up-regulate the expression and secretion of galectin-9 (Gal-9), and promote TIM-3 mediated T cell death (63). At present, there are no relevant studies on how ISGs affects the specific molecular pathways of TEX in SLE patients. This study is the first to screen TEX-related genes from the perspective of TEX, providing a new approach and basis for the treatment of SLE.

GSEA analysis showed that the expression of these biomarkers was significantly positively correlated with immune-related pathways such as NLR signaling pathway, TLR signaling pathway, and RLR signaling pathway. Numerous studies have consistently demonstrated that IFN-I, particularly IFN-α, serves as the principal pathogenic mediator in SLE (64–66). The majority of patients with SLE exhibit overexpression of genes associated with the IFN-I pathway in peripheral blood cells (67–69). Studies have shown that TLR signaling exists in SLE (70, 71). TLRs, especially TLR7 and TLR9, are involved in the activation of plasmacytoid dendritic cells (pDC) to produce IFN-I (72). The increased TLR7 expression is associated with the risk of SLE and the severity of lupus in mice (73, 74). Some studies have shown that RIG-I is significantly expressed in kidney tissue samples from patients with LN, in addition (75). Other studies have demonstrated that the expression level of RIG-I mRNA in urinary sediment is significantly elevated in patients with LN compared to those with IgA nephropathy (76), hinting at a potential role of RIG-I in the underlying pathogenesis of LN. At present, there are few studies on SLE and NLR signaling pathway. All the 4 biomarkers studied in our study were ISGs, which may play a role in SLE by interfering with IFN signaling pathway.

We showed by immune infiltration analysis that naive B cells, resting memory CD4 T cells and resting NK cells exhibited significant downregulation. This is consistent with most of the available findings. Research has demonstrated that immune imbalance is an important factor in the emergence and progression of SLE (77). Studies have shown that the proportion of naive B cells is significantly lower in patients with SLE (78). Most recent studies indicate a significant reduction in the number of NK cells present in the peripheral blood of SLE patients (79–81). However, the underlying cause remains elusive. Cells differentiated from a subpopulation of CD4+ memory T cells may rapidly secrete effector cytokines to promote memory responses to reinfection (31). In the study conducted by Kosalka et al., a notable decrease in the absolute counts of naive CD4+ T cells and T central memory cells within the lymph nodes was observed, with the decrement being particularly pronounced in patients experiencing active disease (82). Currently, the fundamental mechanisms of information transmission and functionality of these cells in SLE remain largely unexplored. Research has demonstrated that SLE patients exhibit abnormal distribution of CD4+ memory T cell subsets, along with a diminished proliferative response to *in vitro* stimulation and a notable elevation in cellular apoptosis (83, 84). However, the results of various studies on memory CD4+ T cells are now inconsistent (85).

Immunoinfiltration analysis showed upregulation of regulatory T (Treg) cells, monocytes, activated dendritic cells and resting mast cells. Increased accumulation of DCs in affected tissues is associated with tissue inflammation and damage (86). In response to microenvironmental stimuli, monocytes can polarize into M1-like or M2-like macrophages. The imbalance between the M1 and M2 phenotypes correlates with SLE pathogenesis, and disease activity (87, 88). Although most reports have concluded that circulating Treg numbers are reduced or functionally impaired (89), some research groups have found elevated Treg levels in patients with SLE that correlate with disease activity (90). These findings are consistent with our immune infiltration results.

Drawing upon our literature review, we postulate that SLE can be triggered by the augmentation of the IFN pathway following the IFN-induced expression of ISGs. Additionally, prolonged exposure to IFN can lead to the expression of STING, IL-10, IDO, PD-L1, TIM-3, LAG-3 and other negative regulatory factors, which induced the apoptosis of T cells. Meanwhile, IFN also triggered alterations in the mitochondrial metabolic pathway of CD8+ T cells, leading to the death and exhaustion of chronically activated CD8 T cells, thereby contributing to the pathogenesis of SLE.

In order to fully understand the potential mechanism of action between the 4 biomarkers and TEX, the TF-mRNA network and ceRNA network were constructed in this study, and the results showed that the 4 biomarkers were regulated by different TFs. FOXC1 and USF2 are co-regulated TFs. At present, the main research on the 8 micrornas suggested in the study focuses on the secretion of cellular inflammatory factors, cell metabolism, apoptosis, proliferation, invasion and migration, especially in tumor (91). However, there are few studies on SLE. LncRNAs are critical in regulating lymphocyte activity, T-helper cell polarization, and adaptive immune cells. BPA, BaP, Tetrachlorodibenzodioxin (TCDD) are not used as drugs, but they are also present in our lives. BPA is an industrial chemical widely used in the polymerization of plastics and in non-food-related materials, such as thermal paper receipts, eyeglasses and children’s toys (92). >90% of human BPA exposure occurs through the oral route, with absorption occurring in the gastrointestinal tract (93). BPA closely resembles the physiological impacts of estrogen (94). BPA induces structural alterations in DNA *in vitro* and is strongly recognized by SLE IgGs and induces high titer antibodies in rabbits (95). Lee et al. demonstrated that the exposure of mouse embryonic stem cells to BPA and progesterone led to a reduction in LY6E mRNA levels, indicating the suppressive effect of these compounds on LY6E expression (96). BPA may act on SLE by interfering with different signaling pathways. BaP is found in smoky environments, processed meat products such as smoked and barbecued. It has been extensively studied and is thought to be associated with an increased risk of many types of cancer and is listed as toxic or dangerous by several countries (97). TCDD is generally considered to be the most toxic synthetic molecule known. TCDD used to be a contaminant of some herbicides and is a harmful contaminant produced by industrial processes (98). Based on the findings of this study, we recommend that patients adopt appropriate preventive measures. These measures include selecting environmentally friendly products free from BPA, avoiding the use of dioxin-containing pesticides and other harmful compounds, and minimizing exposure to environmental pollutants such as industrial emissions, traffic exhaust, waste incineration, barbecuing, and tobacco smoke. Utilizing molecular docking, we predicted chemical compounds that specifically target biomarkers, and in light of the current limited research, we postulate that these chemical compounds have the potential to bind to and disrupt mRNA expression via the interaction with these biomarkers.

In conclusion, based on bioinformatics analysis, we identified 4 biomarkers that were highly correlated with T cell depletion in SLE patients, including LY6E, OASL, MX1, and IFI44. Our findings provide potential therapeutic targets and shed light on the pathogenesis of SLE. However, there are some limitations to our study. First, the patients in the validation cohort were made up of Chinese. Therefore, our findings may not generalize across racial and ethnic groups of SLE patients. Second, there are limited samples available for RT-qPCR validation and insufficient validation has been performed. Therefore, more mRNA expression that would validate these biomarkers is needed. In the forthcoming research undertakings, our principal emphasis will be placed on conducting comprehensive studies encompassing SLE patients from various racial and ethnic backgrounds. These studies will strive to meticulously assess the universal applicability of biomarkers MX1, LY6E, IFI44, and OASL across various global populations. Third, addressing the challenge of insufficient validation resulting from limited sample sizes, we intend to collect samples from SLE patients at various stages of the disease. This will enable a more comprehensive validation of the role these biomarkers play in the initiation and progression of SLE. Simultaneously, we will integrate other methodologies, including gene silencing and overexpression assays, flow cytometry, and immunoimprinting, to explore the molecular regulatory mechanisms underlying the interplay between these biomarkers and T cell exhaustion. Furthermore, we aspire to embark on multi-center, interdisciplinary collaborative research initiatives, ultimately enhancing the quality of life for SLE patients and fostering advancements in this research domain.

## Data Availability

The datasets presented in this study can be found in online repositories. The names of the repository/repositories and accession number(s) can be found below: https://www.ncbi.nlm.nih.gov/geo/, GSE72326 https://www.ncbi.nlm.nih.gov/geo/, GSE81622.
