# Supplementary material for: Identification and immunoassay of biomarkers associated with T cell exhaustion in systemic lupus erythematosus
Source: Front Immunol. 2025 Mar 26;16:1476575. doi: 10.3389/fimmu.2025.1476575 (PMC11979134; doi:10.3389/fimmu.2025.1476575)
Supplement: Supplementary file 1 [file Table1.docx]

| **primer** | **sequences** | |
| --- | --- | --- |
| MX1 F | CAGCTCAGGGGCTTTGGAAT | |
| MX1 R | CCTTGGAATGGTGGCTGGAT | |
| LY6E F | CTGCGTGACTGTGTCTGCTA |  |
| LY6E R | ATTGACGCCTTCTGGGATGG |  |
| IFI44 F | ATTCCCCATCGCTGAAGGAC |  |
| IFI44 R | GGACTTCCTCTAGCAGCGTT |  |
| OASL F | TGAGGCAGGAGCATTTCCAG |  |
| OASL R | CTCCTGAGAACCGTGCCATT |  |
| internal reference-GAPDH F | CGAAGGTGGAGTCAACGGATTT |  |
| internal reference-GAPDH R | ATGGGTGGAATCATATTGGAAC |  |

**Supplementary Table 1**. The PCR primer sequences.
